# Treatment of calcium channel blocker overdose with levosimendan

**DOI:** 10.1186/cc9515

**Published:** 2011-03-11

**Authors:** A Sencan, T Adanır, G Terzi, A Atay, N Karahan

**Affiliations:** 1 Izmir Ataturk Educational and Research Hospital, Izmir, Turkey

## Introduction

We report a case in which cardiovascular collapse after suicidal calcium channel blocker (CCB) overdose was successfully treated with levosimendan with traditional treatment.

## Methods

A 20-year-old male who had taken 250 mg amlodipin besilat was admitted to the ICU from the Emergency Department. His blood pressure was 70/52 mmHg, heart rate 95 bpm and oxygen saturation 99%. An arterial catheter was inserted and arterial blood pressure (ABP) of 52/20 mmHg was measured. He was tracheally intubated and dopamine infusion of 10 μg/kg/minute, dobutamine infusion of 5 μg/kg/minute was initiated. Dopamine and dobutamine infusions were increased to 20 μg/kg/minute and 15 μg/kg/minute, respectively. Despite very high doses of vasopressors, his ABP tended to decrease below 50 mmHg and frequent epinephrine boluses were given. Upon arrival 8 hours later, levosimendan was initiated without an initial loading dose infusion of 0.2 μg/kg/minute. In 4 hours from initiation of levosimendan treatment, dobutamine and dopamine infusions were stopped respectively. After full recovery the patient was discharged 72 hours after arrival.

## Results

CCB overdose causes intractable hypotension, bradycardia, cardiac conduction abnormalities and depression of myocardial contractility, leading to central nervous system, respiratory and metabolic disorders that are often refractory to standard resuscitation methods. Therapy of intoxication includes measures to inhibit further ingestion and absorption with gastric lavage and activated charcoal, to maintain adequate blood pressure with high doses of catecholamine and fluid replacement and to reverse negative inotropic effects by β-adrenergic agonists, phosphodiesterase inhibitors, glucagon, insulin with dextrose and calcium salt. Well-known inotropic agents show their effects via increasing intracellular calcium level. In CCB overdose patients, the efficiency of these drugs was limited because the calcium channels have already been blocked. A new inotropic drug, levosimendan, acts as a calcium sensitizer and increases the association rate of myosin actin cross-bridges and slows down their dissociation rate by binding to troponin C. It also exhibits systemic and coronary vasodilatation via ATP-sensitive potassium channels in vascular smooth muscle cells and on mitochondria.

## Conclusions

We suggest that levosimendan can be considered an additional treatment option in patients with cardiovascular collapse due to CCB intoxication that is refractory to standard management.

